# What Is the Best Regimen for Ovarian Stimulation of Poor Responders in ART/IVF?

**DOI:** 10.3389/fendo.2020.00192

**Published:** 2020-04-17

**Authors:** Zeev Blumenfeld

**Affiliations:** Reproductive Endocrinology, Rappaport Faculty of Medicine, Technion—Israel Institute of Technology, Haifa, Israel

**Keywords:** POF, POI, COH, IVF/ART, poor responders

## Abstract

The infertile patients with aging ovaries—also sometimes referred to as impending premature ovarian insufficiency (POI), impending premature ovarian failure (POF), or poor ovarian responders (POR), constitute a significant and increasing bulk of the patients appealing to IVF/ART. Different causes have been cited in the literature, among the identified etiologies, including chromosomal and genetic etiology, metabolic, enzymatic, iatrogenic, toxic, autoimmune, and infectious causes. Although the most successful and ultimate treatment of POI/POF/POR patients is egg donation (ED), many, if not most, of these infertile women are reluctant to consent to ED upon the initial diagnostic interview, requesting alternative solutions despite the low odds for success. Despite anecdotal case reports, no unequivocal treatment proved to be successful for these patients in prospective randomized controlled trials. Nevertheless, the addition of growth hormone (GH) to ovarian stimulation in POR with GH deficiency may improve the results of controlled ovarian hyperstimulation (COH) and the IVF success. In patients with autoimmune etiology for POR/POI, the combination of glucocorticosteroids, pituitary-ovarian suppression, and COH may be successful in achieving the desired conception.

## Introduction

The infertile patients with aging ovaries—also sometimes referred to as impending primary ovarian insufficiency (POI), impending premature ovarian failure (POF), or poor ovarian responders (POR), constitute a significant bulk of the patients appealing to IVF/ART (1–3). The prevalence of this group of patients seems to be increasing, due to many patients postponing conceptions to the late thirties or even beyond the age of forty. In over half of these patients, no etiologic cause can be pinpointed (1–3). Whereas depletion of most of the ovarian follicles due to older age is well documented, there are several other etiologies associated with poor ovarian reserve (1–3). Among the identified etiologies, different causes have been cited in the literature, including chromosomal and genetic etiology (1, 4–8), and metabolic (4, 9, 10), enzymatic (4, 9, 10), iatrogenic (4, 11), toxic (1–8), autoimmune (1–4), and infectious causes (1, 2, 4–6, 9).

It is beyond our scope to exhaustively elaborate on all the published syndromes and genes associated with POI/POF/POR. Several comprehensive reviews have summarized the genetic etiology, and the list of chromosomal aberrations associated with POI/POF/POR has been increasing in the last decade due to great improvements in genetic technology (1, 12–18).

Although the most successful and ultimate treatment of POI/POF/POR patients is egg donation (ED), many, if not most, of these infertile women are reluctant to consent to ED upon the initial diagnostic inter-view, requesting alternative solutions despite the low odds for success (1–3).

Despite being far from “cracking the code” of successful fertil-ity treatment in POI/POF/POR, many patients feel the need to be convinced that no other solution, except ED, is applicable in their specific case. Only when the alternative solutions prove unsuccessful, as is unfortunately the case in most such attempts, will most POR patients consider and accept the solution of ED.

## Definition and Etiology

Although no unequivocal definition of the poor responders has been universally accepted, the Bologna classification defines poor responders by two of the following characteristics:

- Maternal age 40 years or older, or other risk factors for poor ovarian response (such as excision of bilateral ovarian endometriomas),
- Poor ovarian response in previous IVF cycle(s) (retrieval of three or fewer oocytes in a conventional stimulation IVF protocol), and
- Low antral follicle count (AFC) (less than 5–7 follicles), or low anti-Müllerian hormone (AMH) below 0.5–1.1 ng/ml (3.5–8 pmol/L) (19).

More recently, a new classification of poor ovarian reserve patients in IVF/ART has been put forward by the POSEIDON (Patient Oriented Strategies Encompassing Individualized Oocyte Number) group (20–22).

In this classification, four subgroups have been suggested according to qualitative and quantitative parameters, like the Bologna criteria, namely:

- Age and the expected aneuploidy rate
- Ovarian biomarkers (AFC and AMH), and
- Ovarian response to COS in a previous ART/IVF cycle.

The four POSEIDON classification groups (20) are:

- **POSEIDON group 1**: Patients younger than 35 years old, with normal markers of ovarian reserve (AMH>1.2 ng/mL, AFC>5), and with an unexpected poor ovarian response (POR).
  - ∙ Subgroup 1a: <4 retrieved oocytes on conventional COS in ART/IVF cycle,
  - ∙ Subgroup 1b: 4–9 retrieved oocytes on conventional COS in ART/IVF cycle,
- **POSEIDON group 2**: Patients older than 35 years old, with normal markers of ovarian reserve: AMH>1.2 ng/mL, AFC>5, and with an unexpected poor ovarian response (POR).
  - ∙ Subgroup 2a: <4 retrieved oocytes on conventional COS in ART/IVF cycle,
  - ∙ Subgroup 2b: 4–9 retrieved oocytes on conventional COS in ART/IVF cycle,
- **POSEIDON group 3**: Patients younger than 35 years old, with poor ovarian reserve: AMH <1.2 ng/mL, AFC <5,
- **POSEIDON group 4**: Patients older than 35 years old, with poor ovarian reserve: AMH <1.2 ng/mL, AFC <5.

The POSEIDON classification concept offers a possibly improved stratification for POR patients, which might potentially improve study design and help to fine-tune prognostication. It presents several possible advantages over previously described models, facilitating the evaluation of strategies that could generate higher success of ART/IVF for specific subgroups of patients. It may, in addition, enable the fertility specialist to more accurately advise their patients regarding their treatment prognosis. Indeed, the first relevant indication and confirmation of the low prognosis of POR, stratified according to the POSEIDON criteria, has been recently published (23). In this Dutch multicenter study of 551 poor prognosis patients, cumulative live birth rates (CLBRs) over 18 months and several IVF/ICSI cycles was correlated to the POSEIDON groups (23). They have found about 56% CLBR in poor-prognosis ART/IVF patients over 18 months with variations between the various POSEIDON groups, primarily attributable to the effect of age. Young patients who were classified, according to the POSEDON stratification, as unexpected poor responders (group 1 reached a CLBR of about 65%, whereas the young expected poor responders (group 3) achieved only 59% CLBR (23). In comparison the older unexpected poor responders (group 2) achieved a CLBR of only 42%, and the older expected poor responders (group 4) only 39% (23). For comparison, the CLBR of young normal responders with a normal ovarian reserve was 72% and for the older normal responders it was 58%. The optimistic findings of this study show for the first time that the POSEIDON stratification is correlated to the success rate of ART/IVF, when analyzed jointly with CLBRs (23).

Also, recently, Alviggi et al.(24) have suggested and introduced a new index, called the follicle-to-oocyte index (FOI) for poor responders. Whereas POR is characterized by a reduced number of follicles output rate (FORT), they suggested FOI as a new parameter to characterize POR. The pathophysiologic mechanisms of POR or POI are poorly understood. Furthermore, in over half of such cases there is no explanation. The pathophysiologic mechanisms put forward to explain POR, according to Alviggi et al., is associated with polymorphism of the gonadotropins and their receptors (24). Among the genetic mutations associated with POR were: LH-β subunit variant, G allele carriers of a common FSH receptor (FSHR) polymorphism (p.N680SA > G, rs6166), and other mutations of the FSHR (5–7, 18, 24).

In addition to genotypic polymorphism, these investigators mentioned environmental pollutants and oxidative stress as pathophysiologic factors possibly leading to POR (24).

According to Alviggi et al. (24), FOI might better reflect the dynamic nature of follicular growth in response to COH, compared to the traditional markers of ovarian reserve.

Antibodies to the FSHR or to gonadotropins have been also put forward as possible pathophysiologic causes of POR and POI (1–4), as well as metabolic causes, such as galactosemia (9, 10).

Many POR patients insist on multiple attempts to achieve the desired pregnancy, using their own eggs. Numerous protocols and medications have been put forward to achieve the “gold bullet” which will enable success.

## Possible Treatments

Obviously, the best “treatment” for poor responders after a few IVF failures is egg donation. Whereas the live birth rate (LBR) in POI and POR patients ranges from less than 1 to 10% per cycle, the LBR after egg donation ranges from 50 to 70%. However, most or almost all patients with POI or POR would persist on multiple attempts to achieve the desired pregnancy, using their own eggs. Unfortunately, most of the suggested protocols were not more successful than the previously used protocols in these patients. Among these suggested protocols and medications are:

- GnRH analogues (3, 25, 26)
- Androgens (25)
- GH cotreatment (25, 27)
- Natural cycle or modified natural cycle (25)
- High dose gonadotropins (25)
- Glucocorticoids (3)
- Coenzyme Q10 (28)
- Acupuncture (29)
- Holistic medicine (25)
- Autologous Platelet-Rich Plasma
- *In-vitro* activation of follicles
- Combination of the above (25).

We will briefly and critically address a few of the suggested protocols and associated drugs or cotreatment modalities put forward to improve the results of POR patients in ART/IVF.

## GnRH Analogues

Huang et al. have recently compared the efficiency of the GnRHa vs GnRH antagonist protocols in 1233 POR patients (26). They have found a lower cancellation rate (10 vs. 22%), higher implantation rate (25.3 vs. 10.7%), and higher LBR (27.6 vs. 13%) in young POR patients (POSEIDON group 3), but not in the older POR patients (POSEIDON group 4), undergoing the GnRH agonist COS protocol than in those using the antagonist protocol (26). They concluded that the agonist protocol was more effective than the antagonist protocol for young POR patients (26). However, other recent studies found no difference in cumulative LBR in POR patients according to the Bologna criteria, irrespective of the type of pituitary suppression by GnRH agonists or antagonists (30, 31).

## Androgens

Androgen receptors (AR) are expressed in the theca cells, granulosa cells, and ova (25, 32–34). Expression of AR in follicular cells is critical for normal folliculogenesis and ovulation (25, 32–34). Therefore, various androgens, mainly testosterone and DHEA, have been clinically tried as cotreatment before and during COS in patients with POR but the success was very limited and equivocal. (25, 32–34). Whereas androgens may augment the early stages of folliculogenesis, they may be detrimental, in supraphysiologic concentrations, on the later stages of folliculogenesis, leading to follicular arrest in the sizes of 2–9 mm, inhibiting the formation of a mature Graaffian follicle, as it is in PCOS patients (33–35).

Most recently, a metaanalysis of RCT using testosterone cotreatment in POR patients found that adding testosterone to COS has significantly increased the number of retrieved oocytes, number of generated embrya, clinical PR, and LBR in comparison to controls (32).

A recent review (36) suggests that testosterone may play a beneficial role in folliculogenesis and may be possibly beneficial in COH for POR. However, the evidence from published clinical trials is weak and falls short from drawing robust conclusions regarding the effect of testosterone in POR, since the short administration or the high dose of testosterone are not in keeping with the physiologic role of androgens in the ovary and the presence of androgen receptors during folliculogenesis (36, 37). Indeed, published studies have used inconsistent doses and duration of testosterone in COH. The ongoing T-TRANSPORT trial, for the first time, aims to provide robust evidence regarding the possible beneficial role of transdermal testosterone in COH in ART (36, 37).

Many studies examined the effect of dehydroepiandrosterone (DHEA) supplementation on COS for ART/IVF in patients with POR, with equivocal results (25, 38–40).

Several publications reported on improved hormonal levels, higher number of retrieved ova and generated embrya, better fertilization rates, improved embryo quality, lower cycle cancellation rate, fewer pregnancy losses/miscarriage rate, and higher clinical and cumulative pregnancy rates (25, 38–47).

On the other hand, other studies did not find any improvement in the results of COS in POR patients cotreated with DHEA (25, 48–52). There was no difference in the fertilization rate, no increase in the number of generated embrya, no decrease in the pregnancy losses/miscarriage rate, no more clinical or ongoing PR, and no improvement in LBR (25, 48–52).

It can be concluded, therefore, that the addition of androgens to COS in POR patients does not ubiquitously generate a sensational, or even a significant, improvement in the results of ART/IVF.

## GH Cotreatment

The addition of GH to gonadotropins in COH may up-regulate the intra-ovarian IGF-I, and augment the stimulatory effect of FSH on folliculogenesis (53–58).

Indeed, others (25, 27, 59–61), and we (54–57), have found that GH cotreatment may augment the folliculogenetic effects of gonadotropins and possibly increase the conception rate.

Recently, Kulvinder et al. (62) declared that the addition of GH to COH of POR is an attractive option for increasing pregnancy rates in ART/IVF. Indeed, in several patients this expensive cotreatment in COH might increase PR and LBR. Unfortunately, not in all POR patients. Dakhly et al. (63) examined GH role in a prospective randomized controlled trial (RCT) in 240 Bologna criteria POR. The first had COH with long GnRHa protocol, and the second COH+GH (63). Despite an increase in the number of retrieved oocytes, metaphase II (MII) ova, fertilized oocytes, and transferred embryos, no significant difference was detected in the LBR (63). In another double-blind RCT in POR patients, GH cotreatment did not improve neither follicular recruitment, nor estradiol secretion by mature follicles or the number of retrieved oocytes (64).

The confusion and equivocal results regarding GH cotreatment in IVF and COH is even greater. Several studies have reported on the greater number of overall and MII oocytes (25, 65–67), higher fertilization rates (25, 65, 68), and the increased number of overall generated embryos (25, 67, 69)—top-quality and cryopreserved embryos —in GH cotreatment cycles. On the other hand, other studies reported no difference in the number of overall and metaphase II (MII) oocytes (25, 70–72), no improvement in embryo quality (25, 70, 71), no difference in clinical pregnancy rates (25, 65, 66) and no difference in live birth outcomes (25, 63–65, 72).

Albu and Albu (73) have reported a case of a 29-year-old, GH deficient, infertile patient, who successfully conceived and delivered a healthy boy, on the second IVF cycle after 3 months of GH cotreatment, despite no difference in the number of retrieved ova, compared to the previous, unsuccessful control IVF cycles, without GH. The GH cotreatment improved the eggs' and generated embryos' quality (73). Similarly, three decades ago, we reported on a panhypopituitary patient who failed to conceive on several hMG/hCG COH cycles, and after addition of a very small amount of daily GH, along hMG/hCG, she successfully conceived and gave birth (74). Addition of only 4 units of GH/day (16-24 GH units/cycle) to hMG COH brought about a significant diminution in hMG consumption: 2,700 units/cycle instead of 5,700–7,200 units/cycle (74). The patient conceived on the second cycle of combined GH/hMG/hCG cotreatment and delivered, at term, a healthy neonate (74). Indeed, the synergistic effect of GH and gonadotropins in achieving conception has been proven in infertile patients with GH deficiency, but not in non-GH deficient POR patients (54–57, 74, 75). The addition of GH cotreatment to COH for IVF patients is quite expensive, ranging from 11,400–15,000$/cycle, and 102,000$ overall for achieving a successful delivery (62). It is, therefore, logically and scientifically justified to use this potentially effective but expensive cotreatment only in the GH-deficient POR patients who may clinically benefit from it, by improving the pregnancy rate and “take home baby” rate (54–57, 73, 74).

How can we identify those GH-deficient patients? The clonidine test is a simple test, capable of identifying GH deficient patients or those with very low GH reserve (54–57, 74, 76–78). Based on this simple test, it is possible to prospectively identify those POR candidates who may benefit from GH cotreatment along COH for ART/IVF or (54–57). Whereas 14 pregnancies were successfully generated in 24 clonidine negative patients (58.3%), either in the GH/hMG/hCG cotreatment cycle or in the succeeding one, however GH co-treatment did not generate any pregnancy in eight clonidine positive patients (54, 55).

It can be concluded, therefore, that GH may be beneficial and increase the ovarian response and generated PR and LBR in clonidine negative POR patients but not in clonidine positive infertile patients (54–57, 74, 76).

## Type of COH: Natural/Modified Natural Cycle or High Dose COH

The practice committee of the American Society for Reproductive Medicine (ASRM) has recently summarized and compared the PR for POR patients in ART/IVF in the natural cycle or with mild COH vs. conventional IVF (79).

Similarly, ESHRE consensus has defined the Bologna criteria of “poor response” to COH for IVF. (80). The Bologna criteria of “poor response” to COH for IVF, necessitates the presence of two, or more, of the following three criteria: (1) advanced maternal age or other risk factor(s) for POR; (2) a previous POR on COH for ART/IVF; and (3) an abnormal ovarian reserve test (80). In a more recent review, Busnelli and Somigliana elucidated the possible weaknesses of the Bologna criteria and analyzed the economic aspects of ART/IVF in POR patients (81). Although the Bologna criteria were validated by the available evidence, this review criticized several aspects of the definition, mainly the identified population homogeneity, the chosen cut-off values for the ovarian reserve tests, and the risks factors other than age (81). Similarly, the data regarding the economic profile of poor responders were considered scanty and one study claimed that IVF in POR is not cost-effective, suggesting more studies on this aspect are necessary (81).

The results of two RCTs that compared mild ovarian stimulation vs standard high-dose stimulation IVF in POR patients showed comparable clinical PR (82, 83). On the other hand, POR patients did not benefit from a high starting dose of gonadotrophins in COH for ART/IVF (83).

Berkkanoglu and Ozgur compared daily fixed doses of 300 IU of rFSH, 450 IU, or 600 IU, in a randomized study (84). They found no significant differences in any outcome parameter, such as maximal estradiol levels, number of stimulation days, number of metaphase 2 oocytes, number of transferred embryos, clinical PR, and cancellation rates between the three groups (84). Therefore, increasing the daily FSH dose beyond 300 units increases the cost burden to the patients without additional benefit (84).

Conversely, Ezra et al. (85), reported different results. These investigators retrospectively compared increasing the gonadotropin daily dose from 450 U/day to 300 U twice daily in poor responders (85). They included 23 consecutive poor responders in IVF COH who had previously been treated with 450 U of gonadotropins, followed by an additional IVF cycle using 300 U twice a day, were included (85). This study reported that patients receiving daily gonadotropin 300 IU twice daily reached higher maximal estradiol levels (*P* < *0.03*), higher number of follicles >15 mm in diameter on day of hCG administration (*P* < *0.03*) and more oocytes retrieved (*P* < *0.02*) with 5% live birth rate (85). However, this preliminary report awaits validation by prospective high quality RCTs.

An older, non-randomized study compared minimal stimulation to high-dose COH for POR patients in IVF cycle (86). The clinical PR and the LBR were significantly higher in the mild stimulation protocol compared to the high COH protocol, *P* = *0.007* and *P* = *0.034*, respectively (86).

The ASRM practice committee concluded that in POR patients, there is fair evidence to support the recommendation that mild COH is cost-effective, although LBRs are extremely low and comparable in the natural or modified natural cycle, mild, or conventional COH IVF (79).

Whereas high gonadotropin stimulation cannot compensate for the significantly reduced number of ovarian follicles, another suggested strategy in POR was to exploit the multiple follicular waves within the ovarian cycle, by a double stimulation protocol (follicular and luteal) in the same ovarian cycle (81). Whether this approach yields better results to two cycles of conventional COH is undetermined yet. Another recent publication raised the question of safety and addressed to the double stimulation protocol as “the most intriguing strategy to treat” POR patients in IVF (87).

## Glucocorticoids

In a few cases of POF/POI, and possibly also in POR patients, the etiology seems to be an incorrect immune recognition of ovarian self-antigens, such as anti FSH-receptors, associated with other autoimmune phenomena, and/or antibodies against different tissues besides the ovary (1–4). The association between autoimmune diseases, such as Addison disease and thyroiditis, and POF/POI and anti FSH-receptors antibodies has been documented, and POF/POI may be a part of an autoimmune polyglandular insufficiency including hypoadrenalism, hypoparathyroidism, and mucocutaneous candidiasis (1, 4, 7).

The possible efficiency of treatments such as glucocorticosteroids for immunosuppression, GnRHa, exogenous high-dose gonadotropins, and estrogen replacement is unclear, despite suggestions by many anecdotal case reports (1–4). Furthermore, the beneficial effect of these treatments and a cause–effect relationship has not been demonstrated in prospective RCTs (1–4).

Whereas the prevalence of adrenal autoimmunity in the general population is approximately 1:10,000, it can be found in 2–10% of POF/POI patients (1, 4, 7). It has been postulated that antibodies against the gonadotropin receptors may play a pathophysiologic role in the mechanism of POR and POF/POI (1, 4, 88).

Therefore, several investigators used glucocorticosteroids and GnRHa, together with high-dose gonadotropins, in an attempt to induce ovulation and achieve pregnancies in POR and POF/POI patients (1–4, 7).

The explanatory rationale to this endeavor was that the inactive high endogenous FSH levels cannot induce ovulation, due to the possible anti-FSH receptor antibodies blocking their activation (1–3).

In an attempt to release the possibly decreased FSH receptors from their occupancy by the endogenous high FSH, or alternatively, to prevent the downregulation of FSH receptors by the very high FSH concentrations, the high FSH levels should be suppressed by GnRHa or estrogen-progesterone pills, in addition to glucocorticosteroids, as immunosuppressors, as well as administration of exogenous gonadotropins to stimulate the released FSH receptors (1–3). Hypothetically, in such a gonadotropin-resistant ovary, or POR, where folliculogenesis may be impeded, removing the block exerted by downregulation of the FSH receptors by the chronically increased FSH levels may restore ovulation once the receptors and follicles return to being responsive to FSH (1–3). In the presence of possible autoimmunity, such as anti-ovarian or anti-FSH receptor antibodies, the administration of low dose glucocorticosteroids may diminish the autoimmune process and possibly lower the level or activity of these antibodies (1–3).

Although numerous case reports have described the return of ovarian function after using immunosuppressive therapies, the lack of an exactly defined criterion for the diagnosis of autoimmune mechanisms and the absence of level I proof for the effectiveness of this endeavor makes such an attempt equivocal (1–3, 88–96). No randomized controlled studies with immunologic monitoring have been performed that could establish the success of this therapy (1–4, 88). Anecdotal, sporadic successes and even a few pregnancies have been generated by glucocorticoids cotreatment, but no prospective RCT could unequivocally support these successful case reports (1–3, 88–96). Interestingly, all the achieved pregnancies by the glucocorticoids/GnRHa/COH occurred in the first three such attempts (1–3). Therefore, if pregnancy cannot be generated within three such attempts, it is recommended to discourage POR patients from further similar COH and proceed with the more successful egg donation (ED) (1–4, 7, 88).

Despite the previously written, in a prospective RCT, 58 POF/POI POR patients with normal karyotype have undergone COH with GnRHa and glucocorticosteroids or placebo (97). Almost 20.7% of the patients in the dexamethasone group successfully ovulated vs. only 10.3% in the placebo group, and two singleton pregnancies were generated in the glucocorticosteroids treated POR patients (97).

## Coenzyme Q10

Whereas the causes of POR are unknown in most cases, and since oxidative stress and mitochondrial dysfunction have been put forward as one of the possible pathophysiological mechanisms, the antioxidant coenzyme Q10 (CoQ10) has been tried as a COH cotreatment in such young patients (98). The CoQ10 antioxidant is a lipid-soluble coenzyme obligatory structure of the inner mitochondrial membrane (98). This coenzyme enables for the electron transport in mitochondrial respiration and oxidative phosphorylation necessary for adenosine triphosphate (ATP) production (98). Although shown to be beneficial in treating male oligo-asthenospermia and in cardiology, the clinical use in POR is not abundant (98–101). Preclinical studies in animals have suggested CoQ10 can protect ovarian reserve, possibly counteracting the physiological ovarian aging by restoring mitochondrial function and augmenting embryo cleavage and blastocyst generation (102–104). In female infertility, CoQ10 supplementation to COH improved patients' response to ovulation induction and decreased fetal aneuploidy in older patients, between age 35 and 43 (105, 106).

In an RCT of 186 consecutive POR patients stratified according to the POSEIDON classification group 3, the participants were randomized to either CoQ10 pre-treatment for two months before COH for ART/IVF vs COH without CoQ10 as controls (98). More oocytes were retrieved in the CoQ10 group, the fertilization rate and the number of high-quality embryos was higher *(P* < *0.05*) (98). The number of patients with canceled ET due to poor embrya was lower (*P* = *0.04*), and the number of patients with available cryopreserved embryos was higher (*P* = *0.012*), in the CoQ10 group vs controls (98). However, the clinical PR and LBR/ET did not reach statistical significance despite a tendency to be higher in the CoQ10 group (98).

Whether CoQ10 supplementation will revolutionize COH protocols for treating POR patients is premature to conclude, and additional prospective RCTs are needed to answer this question.

## Acupuncture

The effects of acupuncture on ART/IVF outcomes are equivocal (29, 107). Most POR patients feel anxiety and frustration after having undergone several unsuccessful IVF cycles. In desperation, they fall back on anything that may possibly improve the outcome of ART/IVF (29). Acupuncture has gained popularity among the various complementary modalities and drugs suggested as cotreatment and which might increase the IVF success (29). A recent metaanalysis of 27 studies including 6116 patients has found that although the clinical PR of the patients who had acupuncture during IVF was significantly higher compared controls (RR 1.21, 95% CI: 1.07–1.38), the LBR was not different (107). Interestingly, subgroup analysis demonstrated that the benefit of acupuncture was more significant for women who had undergone repeated IVF cycles, possibly including patients with POR (29). However, the authors themselves declared that the reporting of the existing studies was poor and they had methodological flaws (101). Therefore, larger studies with better methodologies are needed to validate the findings of their meta-analysis (107).

## Holistic Medicine

The same feeling of anxiety and frustration after POR and repeated unsuccessful IVF cycles led couples to try holistic medicine as they had with acupuncture (25). Holistic health care considers all therapeutic experiences. It has been suggested that comprehensive medical services are needed, in addition to psychological support, counseling, and education (108). It has been emphasized that all patients' questions should be patiently and thoroughly addressed, in order to minimize anxiety and fear and recognize psychological issues that may influence therapy (108). Unfortunately, sometimes, recommendations given by alternative medicine practitioners may contradict or interfere with the instructions given by their IVF physicians (25). Using several nutritional supplements has been cited to induce beneficial effects such as a “more natural” or holistic approach and helping the patients feel more “in control” (25, 109–111).

The use of holistic, alternative, and complementary medicine, mainly by women has turned popular in the Western society (111). Unfortunately, there is no good information regarding the ability of holistic, alternative, and complementary medicine to improve fertility (111).

A systematic review of multiple databases included eight publications on holistic, alternative, and complementary medicine for treating infertility (111). However, this review found significant gaps in the evidence regarding women's use of holistic, alternative, and complementary medicine for fertility enhancement or the success of this approach (111). The authors of this review concluded that comprehensive population-based studies are necessary to substantiate evidence, prevalence, and recommend policy and clinical practice (111).

Until then, no solid evidence exists to recommend holistic, alternative, and complementary medicine for enhancing fertility in women with POR.

Nevertheless, empathic counseling and support, before and during ART/IVF treatment, may be beneficial for every infertile couple, and especially in patients with POR, by ameliorating the treatment associated anxiety and distress (25).

## Autologous Platelet-Rich Plasma

In the last year a “glimpse of new hope” for POR-IVF patients has been suggested by two preliminary publications (112, 113). In these reports, autologous platelet-rich plasma (PRP) was injected intraovarially by transvaginal sonographic guidance, before the IVF COH (112, 113). The preliminary results suggest a trend toward better implantation rates and LBRs in those POR patients who have received the intraovarian PRP injections (112, 113). Interestingly and encouraging, autologous FSH decreased and AMH increased following the PRP treatment (113). However, the number of patients used in these publications is insufficient to draw robust conclusions, and additional studies, preferably RCTs, are awaited to validate this preliminary and optimistic hope.

## *In vitro* Activation of Follicles

Most recently, Kawamura et al. (114) reported on drug-free *in-vitro* activation (IVA) of follicles for infertility treatment in POR patients with DOR. The IVA method suggested a possible infertility treatment for patients with POI (114–117). The IVA approach promotes growth of residual ovarian follicles following ovarian tissue fragmentation leading to Hippo signaling disruption, together with *in-vitro* incubation with follicle activating stimulators (114–117). However, the IVA method has been considered equivocal regarding its efficacy and safety whereas *in vitro* studies have suggested that activation by pharmacological methods may negatively affect oocytes' quality (118–121). Indeed, it has been suggested that IVA combined with PI3K/Akt and Hippo signaling pathways before ovarian slices auto-transplantation may bear major negative consequences on follicle health (118, 122–125).

On the other hand, IVA without the use of pharmacological activation of follicles may be possibly effective and not detrimental to the ovarian follicles (114). As an extrapolation of the IVA approach, Kawamura et al. (114) tested whether Hippo signaling disruption alone using *in-vitro* ovarian cortical fragmentation without *in vitro* stimulation with Akt stimulators, followed by autologous grafting, was sufficient to promote follicle growth. The results of this preliminary study were encouraging. Increased AFC's were observed in 9/11 such treated POR patients (114). Moreover, the metaphase II oocytes number increased from 1 to 2.6, 68.7% of these oocytes were fertilized, and 56.9% generated high-quality embryos (114). Furthermore, one patient naturally conceived, and 16 ETs in 5 patients yielded four pregnancies: one live birth, two ongoing, and one miscarriage (114). In addition, a few patients had cryopreserved embrya (114). These encouraging results await the validation by prospective RCT's.

## Conclusion

Although frustrating to both patients and healthcare practitioners, no solid data recommend on any “magic bullet” protocol for patients with POR. The only protocol offering very high success rate is ED. Unfortunately, many, if not most POR patients will undergo numerous unsuccessful IVF attempts before falling back on the recommended ED.

Despite the above conclusion, two exceptions may justify additional attempts, before ED:

1. In POR patients with borderline GH deficiency (Clonidine negative patients), the addition of GH to COH may improve IVF results.
2. In POR patients with evidence of autoimmunity to various glands and organs (thyroid, adrenal…), suggesting an autoimmune pathophysiology to their POR, a protocol combining glucocorticoids, long GnRHa, and high dose gonadotropins may improve the number of retrieved oocytes, and possibly also the IVF results. Even in cases where there was a significant increase in the yield of generated ova and embrya by this protocol, the maximal recommended attempts is three—since all the pregnancies achieved by using this combination were successful within three attempts (1–3).

In addition, the preliminary optimistic reports on autologous PRP intraovarian injection, and on IVA in POR patients await validation by future prospective RCTs.

## Author Contributions

The author confirms being the sole contributor of this work and has approved it for publication.

## Conflict of Interest

The author declares that the research was conducted in the absence of any commercial or financial relationships that could be construed as a potential conflict of interest.
